# Supplementary figures and images for: Clinical and virological characteristics of hospitalised COVID-19 patients in a German tertiary care centre during the first wave of the SARS-CoV-2 pandemic: a prospective observational study
Source: Infection. 2021 Apr 22;49(4):703–14. doi: 10.1007/s15010-021-01594-w (PMC8061715; doi:10.1007/s15010-021-01594-w)

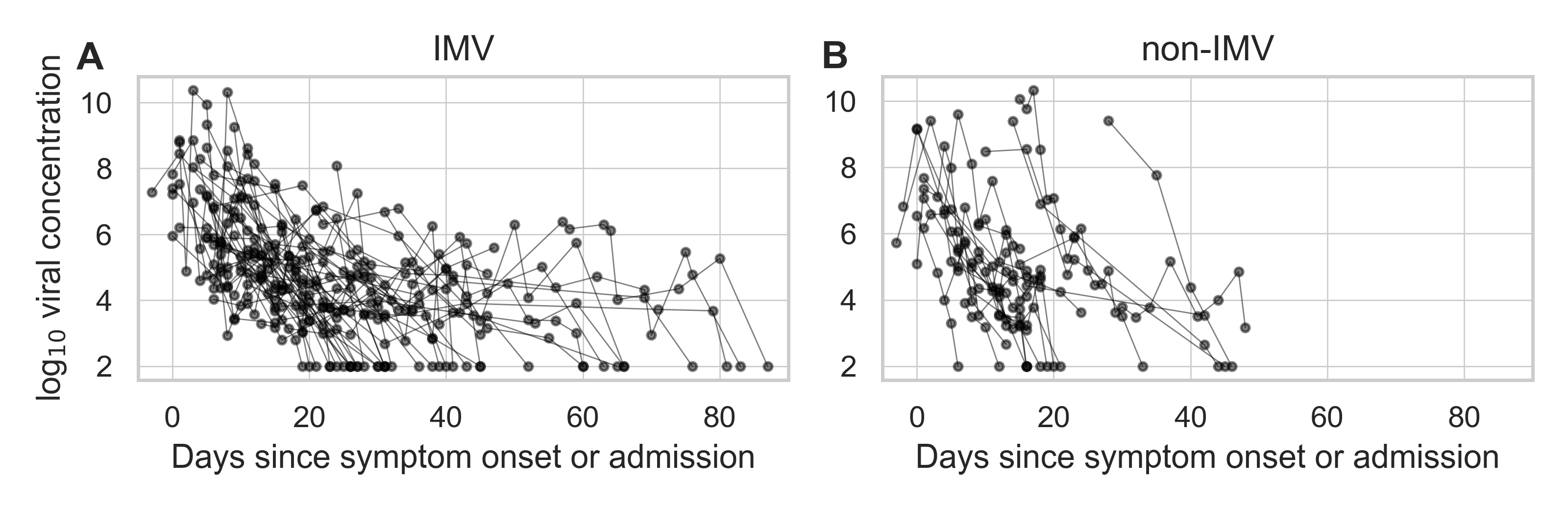

Supplement: Supplementary file 1 — Supplementary Figure 1 Courses of viral concentration over time for A) IMV and B) non-IMV patients. Only patients with viral concentration measurements on at least four different days were included. If a patient had multiple viral concentration measurements on the same day, only the highest viral concentration was included for that day. If available, the first of at least two final negative PCRs is included with a viral concentration of 2.0 assigned. The x-axis displays the number of days since symptom onset, if available (n=63), or number of days since admission (n=10) [file 15010_2021_1594_MOESM1_ESM.tif]

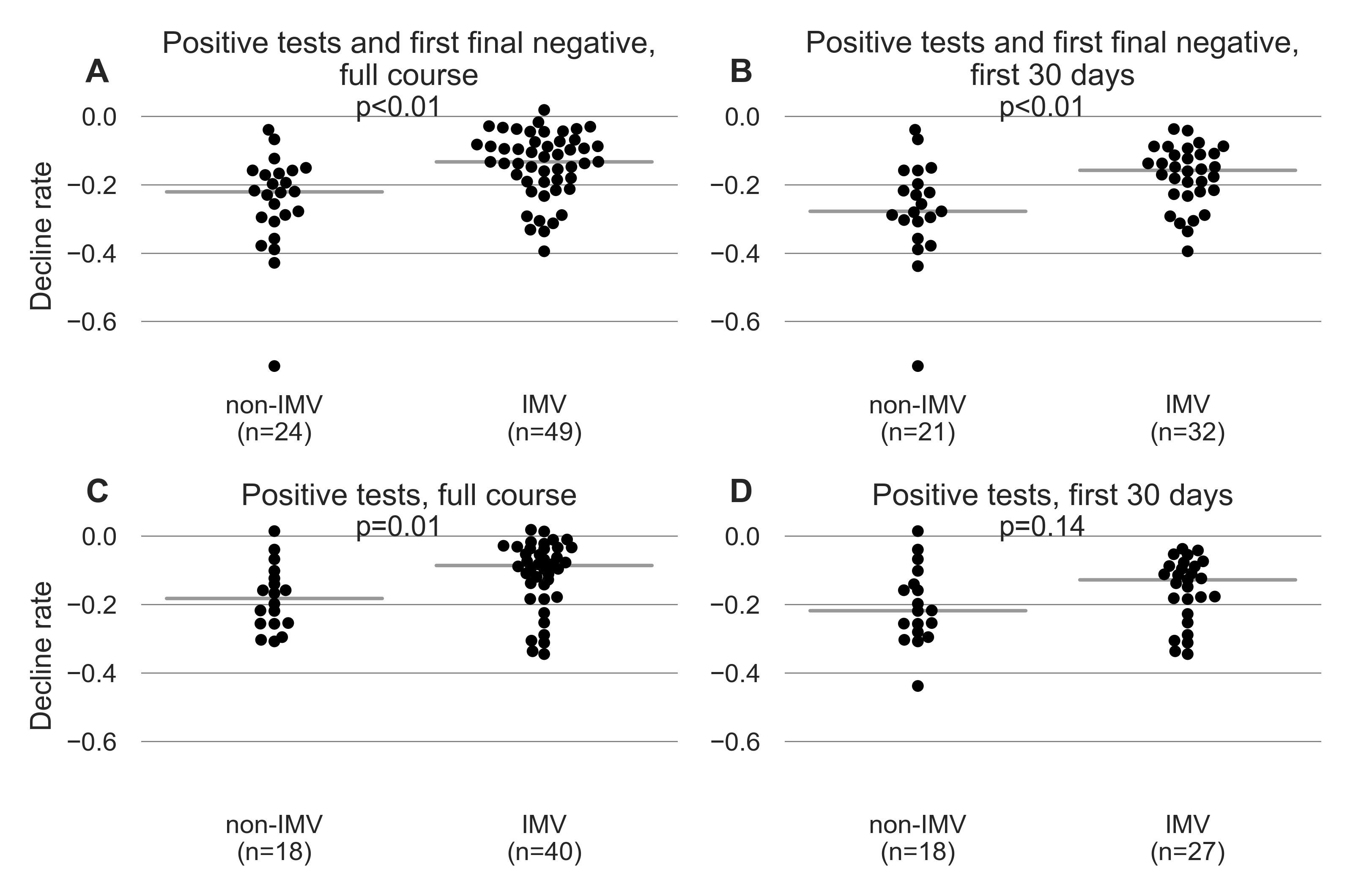

Supplement: Supplementary file 2 — Supplementary Figure 2 Viral concentration decline rates, taking into account full and partial viral concentration courses for each patient. Decline rates were calculated using a linear regression for patients with at least four RT-PCR results. A, B) include positive RT-PCR results and the first of at least two final negative RT-PCR tests for a patient. C, D) only include positive RT-PCR test results. A, C) are based on all RT-PCR test results for a patient, while B, D) only take into account results from RT-PCR tests performed within 30 days of symptom onset (n=63), or date of admission (n=10) if date of symptom onset was unknown. Pairwise comparisons were performed using a Mann-Whitney U test. Grey horizontal lines indicate the median [file 15010_2021_1594_MOESM2_ESM.tif]

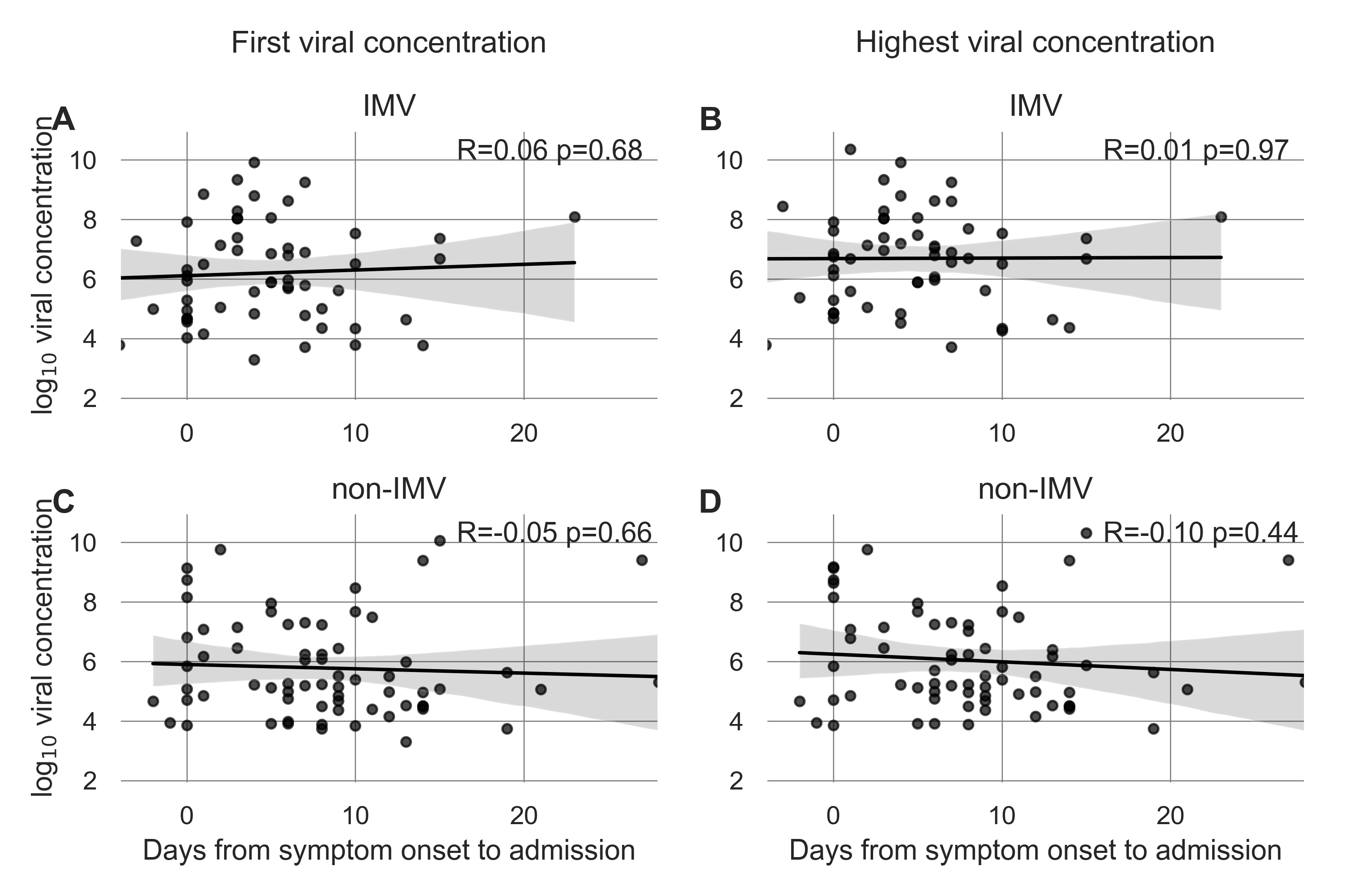

Supplement: Supplementary file 3 — Supplementary Figure 3 Log10 viral concentration plotted against time in days from symptom onset to admission. A) and B) include data from invasive mechanically ventilated (IMV) patients, and C) and D) from non-IMV patients. A and C) show the first-measured viral concentration per patient, and B and D) illustrate the highest viral concentration per patient. Shaded areas indicate the 95% confidence interval. p—p value, R—correlation coefficient [file 15010_2021_1594_MOESM3_ESM.tif]
