# Supplementary material for: Clinical and virological characteristics of hospitalised COVID-19 patients in a German tertiary care centre during the first wave of the SARS-CoV-2 pandemic: a prospective observational study
Source: Infection. 2021 Apr 22;49(4):703–14. doi: 10.1007/s15010-021-01594-w (PMC8061715; doi:10.1007/s15010-021-01594-w)
Supplement: Supplementary file 6 — Supplementary file6 (DOCX 26 KB) [file 15010_2021_1594_MOESM6_ESM.docx]

**Supplementary table 3**: **Antiviral, disease modifying, and immunosuppressant drugs and biologics**. Overview of percentages of treated patients, dose and indication used in this cohort.

| **Drug** | **Percentage, n/N  treated patients** | **Daily dose and route of administration** |  | **Indication  and duration** |
| --- | --- | --- | --- | --- |
| Prednisolon equivalent ≥40mg/day | 10.1%, 17/168 | 100- 250 mg once i.v._a_ |  | Allergic reaction (n=2)  Glottis edema (n = 1) |
|  |  | 100 mg i.v. ≥1 day |  | Fibroproliferative ARDS_b_ (n = 2)  Cryptogenic organizing pneumonia (n = 1)  Obstruction (n = 1)  Stridor post extubation (n = 1)  Not evaluable (n = 1) |
|  |  | 80 mg  i.v. ≥1 day |  | Not evaluable (n = 2) |
|  |  | 50 mg i.v. ≥1 day |  | Fibroproliferative ARDS (n = 2)  Giant cell arteritis (n = 1)  Obstruction (n = 1) |
|  |  | 40 mg i.v.  ≥1 day |  | Acute exacerbation of COPD_c_ (n = 2) |
| Dexamethason | 9.5%, 16/168 | 6 mg p.o._d_ ≥1 day |  | SARS-CoV-2_e_ (n = 13) |
|  |  | 20 mg i.v. ≥1 day |  | Suspected hemophagocytic lymphohistiocytosis (n = 1) |
|  |  | 22 mg i.v.  ≥1 day |  | Suspected hemophagocytic lymphohistiocytosis (n = 2) |
| Remdesivir | 3.6%, 6/168 | 100-200 mg p.o. |  | SARS-CoV-2 |
| Lopinavir/Ritonavir |  | 200/50 mg p.o. |  | HIV_f_-Infection (n = 1) |
|  |  | 800/200mg p.o. |  | SARS-CoV-2 (n = 1) |
| Hydroxychloroquine | 1.2%, 2/168 | 400mg p.o. |  | Systemic lupus erythematosus (n = 1) |
|  |  | 400mg p.o. |  | SARS-CoV-2 (n = 1) |
| Tocilizumab | 0.6%, 1/168 | 162mg i.v. |  | SARS-CoV-2 |
| Anakinra | 1.8%, 3/168 | 200 mg i.v. |  | Suspected hemophagocytic lymphohistiocytosis  (n = 1) |
|  |  | 100 mg  i.v. |  | Suspected hemophagocytic lymphohistiocytosis  (n = 1) |
|  |  | 200 mg s.c. |  | Suspected hemophagocytic lymphohistiocytosis  (n = 1) |
| Immunoglobulins | 1.8%, 3/168 | 10 g i.v.  (cumulative dose 40g i.v.) |  | Suspected hemophagocytic lymphohistiocytosis  (n = 2) |
|  |  | 10g i.v.  (cumulative dose 50 g i.v.) |  | Suspected hemophagocytic lymphohistiocytosis  (n = 1) |

1. i.v. – intravenously
2. ARDS – acute respiratory distress syndrome
3. COPD – chronic obstructive pulmonary disease
4. p.o. – per os
5. SARS-CoV-2 - Severe Acute Respiratory Syndrome Coronavirus 2
6. HIV- human immunodeficiency virus
