# Supplementary material for: Clinical and virological characteristics of hospitalised COVID-19 patients in a German tertiary care centre during the first wave of the SARS-CoV-2 pandemic: a prospective observational study
Source: Infection. 2021 Apr 22;49(4):703–14. doi: 10.1007/s15010-021-01594-w (PMC8061715; doi:10.1007/s15010-021-01594-w)
Supplement: Supplementary file 7 — Supplementary file7 (DOCX 22 KB) [file 15010_2021_1594_MOESM7_ESM.docx]

**Supplementary table 4**: **Laboratory parameters from first 72h after admission** in all, non-IMV and IMV patients. Data from all available n/N are shown as medians and IQRs. Bold indicates statistical significance.

|  | **All** | **Non-IMV** | **IMV** |  |
| --- | --- | --- | --- | --- |
| **Parameter** | **median (IQR), available n/N** | **median (IQR)** **available n/N** | **median (IQR)** **available n/N** | **p-value**  **Mann-Whitney *U* test** |
| CRP mg/L | 68.2 (25.1-124.1), 117/168 | 50.8 (14.7-103.1), 77/90 | 138 (68.2-372.7), 35/71 | **<0.01** |
| PCT µg/L | 0.1 (0.06-0.25), 114/168 | 0.08 (0.05-0.13), 74/90 | 0.27 (0.11-0.85), 35/71 | **<0.01** |
| LDH U/L | 341 (285-460), 114/168 | 303 (263-392), 75/90 | 469 (360.5-622.8), 34/71 | **<0.01** |
| IL-6 ng/L | 48.7 (18-118.6), 82/168 | 27.1 (10.7-72.7), 59/90 | 151.5 (79.8-151.5), 20/71 | **<0.01** |
| Ferritin µg/L | 651.8 (311.4-1640.8), 89/168 | 543.7 (244-1008.4), 57/90 | 1384.5 (524.9-2063.9), 29/71 | **<0.01** |
| WBC /nL | 6.5 (4.9-9.2), 117/168 | 6.04 (3.4-7.8), 77/90 | 7.86 (6-11.7), 35/71 | **<0.01** |
| Lymphocytes /nL | 0.9 (0.65-1.32), 114/168 | 1.01 (0.7-1.4), 75/90 | 0.8 (0.6-1), 34/71 | **0.02** |
| Neutrophils /nL | 4.6 (3.1-6.3), 105/168 | 4.2 (2.7-5.8), 74/90 | 5.57 (4.1-8.9), 27/71 | **<0.01** |
| NLR | 4.6 (3.0-7.0), 105/168 | 4.18 (2.7-5.8), 74/90 | 6.6 (4.2-10.721), 27/71 | **<0.01** |
| Hb g/dl | 13.3 (11.6-14.4), 117/168 | 13.6 (11.9-14.5), 77/90 | 12.7 (11.2-13.9), 35/71 | 0.08 |
| Platelets /nL | 198 (142-266.5), 117/168 | 201 (151.5-277.5), 77/90 | 194 (139-253), 35/71 | 0.68 |
| Creatinine mg/dL | 0.9 (0.8-1.3), 117/168 | 0.88 (0.8-1.1), 77/90 | 1.17 (0.9-1.6), 35/71 | **<0.01** |
| Urea mg/dL | 33.5 (22-51.8), 100/168 | 26 (19-39.5), 62/90 | 41 (31-75.5), 33/71 | **<0.01** |
| Lactate mg/dL | 12 (9-15), 99/168 | 12 (8-14), 59/90 | 13 (10-15), 35/71 | 0.10 |
| Total bilirubin mg/dL | 0.47 (0.31-0.69), 116/168 | 0.45 (0.27-0.65), 76/90 | 0.52 (0.37-0.77), 35/71 | 0.09 |
| ALAT U/L | 31 (20-45), 117/168 | 32 (19-46), 77/90 | 30 (20-43), 35/71 | 0.92 |
| ASAT U/L | 43 (30.5-70.5), 97/168 | 38 (28-55), 59/90 | 59 (40.5-91.6), 33/71 | **<0.01** |
| CK U/L | 95 (56.5-241), 101/168 | 83 (56-132), 67/90 | 260.5 (82.3-700.5), 30/71 | **<0.01** |
| NT-proBNP pg/mL | 202.5 (86-951.3), 106/168 | 136 (42-406), 69/90 | 672.5 (193.8-1815.3), 32/71 | **<0.01** |
| Troponin T ng/L | 12.5 (6-35.5), 74/168 | 7 (5.5-19), 49/90 | 23 (10.5-66), 21/71 | **<0.01** |
| D-Dimers mg/L | 1.8 (0.9-3.2), 43/168 | 0.97 (0.6-2.1), 13/90 | 2.17 (1-3.7), 27/71 | **0.02** |

*IMV* invasive mechanical ventilation, *IQR* interquartile range, *CRP* C-reactive protein, *PCT* Procalcitonin, *LDH* lactate dehydrogenase, *IL-6* Interleukin 6, *WBC* white blood cell count, *NLR* neutrophil-to-lymphocyte-ratio, *Hb* Hemoglobin, *ALAT* alanine aminotransferase, *ASAT* aspartate aminotransferase, *CK* creatine kinase, *NT-proBNP* N-terminal prohormone of brain natriuretic peptide.
